# Isolation and functional characterisation of lamina propria leukocytes from helminth-infected, murine small intestine

**DOI:** 10.1016/j.jim.2019.112702

**Published:** 2020-02

**Authors:** Holly C. Webster, Anna T. Andrusaite, Amy L. Shergold, Simon W.F. Milling, Georgia Perona-Wright

**Affiliations:** Institute of Infection, Immunity and Inflammation, University of Glasgow, 120 University Place, Glasgow G12 8TA, UK

**Keywords:** Helminth, Th2, Small intestine, Cell isolation, T cells, Macrophages, LP, lamina propria, Th2, T helper cell 2

## Abstract

The use of helminth infections as tools to understand the type 2 immune response is a well-established technique and important to many areas of immunological research. The phenotype and function of immune cell populations at the site of infection is a key determinant of pathogen clearance. However, infections with helminths such as the murine nematode *Heligomosmoides polygryrus* cause increased mucus production and thickening of the intestinal wall, which can result in extensive cell death when isolating and analysing cells from the lamina propria (LP). Populations of larger immune cells such as macrophages and dendritic cells are often trapped within mucus or dying tissues. Here we describe an optimised protocol for isolating LP leukocytes from the small intestine of *H.polygyrus* -infected mice, and we demonstrate phenotypic and functional identification of myeloid and CD4^+^ T cell subsets using cytokine staining and flow cytometry. Our protocol may provide a useful experimental method for the immunological analysis of the affected tissue site during helminth infections.

## Highlights

• An optimised protocol for the isolation of lamina propria leukocytes from the small intestine of helminth-infected mice.
• Allows for high yield and viability of immune cells from the infected tissue, despite mucus and inflamed intestinal wall.
• Intracellular cytokine staining on CD4^+^ T cells shows clear Th2 induction in the lamina propria.
• Macrophages, monocytes, dendritic cells and neutrophils also successfully isolated from the helminth-infected small intestine.

## 1 Introduction

A type 2 immune response is established in response to stimuli such as allergens and helminth parasites. The type 2 immune response is characterised by infiltration of innate cells such as basophils, eosinophils and mast cells to mucosal sites, hyperplasia of goblet cells and a robust T helper 2 (Th2) cell response. The type 2 response is often associated with diseases such as allergy and asthma (Yazdanbakhsh et al., 2002), where the type 2 response is inappropriate. However, the type 2 immune response is critical for host protection during helminth infection and expulsion of parasites (Urban Jr. et al., 1991). Therefore, understanding the type 2 response and its regulation is important for fundamental science and for the development of new therapies.

Murine helminth infections are widely used as models of type 2 immune activity, regulation and disease (Gause et al., 2013). A commonly used helminth is the nematode *Heligomosmoides polygyrus*, which naturally infects the small intestine of wild mice. This parasite is in the same family of pathogens as the human hookworms *Necator americanus* and *Ancylostoma duodenale*, although its life cycle is more similar to ruminant parasites such as *Haemonchus contortus (*Reynolds et al., 2012*).* It is often used as a model of a physiological Th2 response during infection*. H. polygyrus* larvae are ingested orally at the L3 stage and, within 24 h, reach the small intestine of their host and begin to migrate through the intestinal wall. Larvae migrate to the submucosa and encyst, undergoing two moults and developing into adult worms. Adult worms then migrate back through the intestinal wall and emerge into the lumen, where they mate and produce eggs (Monroy and Enriquez, 1992). The migration of larvae and adult worms through the small intestine wall results in epithelial cell damage. Damaged epithelial cells release cytokine alarmins such as IL-33, thymic stromal lymphopoietin (TSLP) and IL-25 (Barlow and McKenzie, 2011). IL-33 and IL-25 activate type 2 innate lymphoid cells (ILC2), which will in turn produce type 2 cytokines such as IL-5 and IL-13 (Barlow and McKenzie, 2011). Other innate cells including mast cells, eosinophils and basophils are rapidly recruited to the site of infection and also secrete IL-4, IL-5 and IL-13 (Davoine and Lacy, 2014; Gessner et al., 2005; Mukai et al., 2018). Macrophages and dendritic cells contribute to inflammation and stimulate the activation of Th2 cells, a subset of CD4^+^ effector T cells which are key to the type 2 immune response in helminth infection, allergy and asthma (Kim and Kim, 2018). Th2 cells produce the type 2 cytokines IL-5, IL-13 and IL-4. IL-4 drives further Th2 differentiation and guides class switching of B cells, while IL-5 and IL-13 are predominantly effector cytokines, recruiting and activating granulocytes, promoting degranulation, and guiding wound repair (Allen and Wynn, 2011; Liang et al., 2011). IL-13 stimulates goblet cell hyperplasia, which increases goblet cell production of mucin. Mucus production by goblet cells is a key component of helminth expulsion (Anthony et al., 2007).

One of the most common time-points currently investigated during *H.polygyrus* infection is fourteen days post infection, as Th2 cell expansion peaks (Perona-Wright et al., 2010a), adult worms have emerged into the lumen (Hewitson et al., 2011) and both innate and adaptive immune responses are well established (Rolot and Dewals, 2018). At this time-point, there is large immune cell infiltration to the tissue, and further thickening occurs as a result of fibrosis. Mucus production is high due to goblet cell hyperplasia. Both factors contribute to extensive cell death, experimentally, when isolating lamina propria (LP) leukocytes from the small intestine for further analysis. Published studies that aim to investigate the small intestine LP therefore typically concentrate on earlier time-points or on cells located in lymphoid tissues (Mosconi et al., 2015; Pelly et al., 2016; Perona-Wright et al., 2010a). Furthermore, larger immune cells such as macrophages frequently become trapped in mucus and dying tissues and are therefore very difficult to isolate. The ability to investigate immune cell populations at the site of infection is becoming increasingly important, as interactions between immune cells in inflamed tissues is emerging as key to fully understanding an immune response to infection. We have therefore optimised a method for isolating viable LP leukocytes from the small intestine LP during *H.polygyrus* infection and we show, using cytokine staining and flow cytometry, that this protocol enables the functional identification of subsets of both the myeloid and CD4^+^ T cell compartments.

## 2 Methods

### 2.1 Mice and infection

Seven-week-old female C57BL/6 mice were purchased from Envigo (Huntingdon, UK). Animals were maintained in individually ventilated cages under standard animal house conditions at the University of Glasgow and procedures were performed under a UK Home Office license (Project number 70/8483) in accordance with UK Home Office regulations following review by the University of Glasgow Ethics Committee. Mice were acclimatised for 1 week after arrival in the animal unit, and then infected with 200 *H. polygyrus* L3 larvae by oral gavage.

### 2.2 Isolation of lamina propria leukocytes

Naïve and infected animals were euthanised using carbon dioxide, and the small intestine removed by cutting below the stomach and above the caecum. Care was taken to ensure as much fat as possible was removed from the exterior of the intestine. Intestines were transferred immediately onto laboratory tissue paper soaked liberally in phosphate-buffered saline (PBS) (no calcium, no magnesium; kept at room temperature) and Peyer's patches were removed using dissecting scissors, working quickly to ensure the tissue did not dry out. Continuing on PBS-soaked tissue paper and adding more PBS as needed to keep the tissue wet, the intestines were opened longitudinally using blunt tip scissors and then held by forceps and washed vigorously by agitation in a PBS-filled petri dish to remove intestinal content. Fine forceps were used to gently squeeze out any remaining mucus, running the forceps down the length of each intestine. Each intestine was then transferred onto a fresh piece of PBS soaked tissue and cut into 1 cm pieces. The pieces were collected and transferred to a 50 ml centrifuge tube containing 30 ml of HBSS (Gibco™14,170,088 no calcium, no magnesium) supplemented with 10% FCS (Gibco™ Fetal Bovine Serum, qualified, heat inactivated, E.U.-approved, South America Origin) and kept on ice. Each tube was shaken vigorously by hand and placed on ice for transfer from the animal unit back to the laboratory. Samples should be kept on ice for no longer than 1 h at this stage.

Further processing of the intestines was staggered, depending on sample number: a maximum of 8 samples was processed at one time and each step and wash was carried out quickly, ensuring that the intestinal tissue did not dry out at any stage in the protocol. Each sample was poured onto a large piece of 50-μm Nitex mesh, folded into a funnel placed in a 400 ml beaker. Samples were washed by pouring 30mls pre-warmed HBSS (Gibco™ 14,170,088 no calcium, no magnesium as before, but with no supplements), over the Nitex mesh in the funnel. Using forceps, the samples were then transferred back into tubes containing 15 ml 2 mM EDTA (UltraPure™ 0.5 M EDTA, pH 8.0 Cat. 15,575,020) in HBSS, pre-prepared and warmed to 37 °C). The samples were shaken vigorously by hand and placed into an orbital shaker (Stuart, Orbital Incubator SI500) set to 220 rpm and 37 °C for 15 mins.

After shaking for 15 min, the tubes were removed, the samples poured onto Nitex mesh arranged in a funnel and washed with non-supplemented HBSS exactly as previously. After washing, the intestinal pieces were again transferred to tubes containing 15 ml 2 mM EDTA in HBSS and returned to the orbital shaker for a further 15 min. This process of EDTA washes was repeated twice more, for a total of three 15 min shakes in 15 ml 2 mM EDTA in HBSS. After the 3rd and final EDTA wash, the intestinal pieces were transferred into 15 ml of RPMI 1640 (Gibco™ no glutamine, 21870076) supplemented with 10% FCS, 10% FCS, 100 U/ml Penicillin, 100μg/ml Streptomycin, 2 mM l-glutamine (Life Technologies 15,140,122) and 62.5 CDU/ml Collagenase VIII (CDU, collagenase digestion units) (Sigma-Aldrich C2139-500MG), all pre-prepared and warmed to 37 °C. The samples were shaken vigorously by hand and placed back into the shaker set to 220 rpm and 37 °C. After 10 min, the samples were checked by removing from shaker and shaking vigorously by hand. At this stage, tissue had begun to break up and the medium looked cloudy, reflecting cells released into the supernatant. The samples were placed back into the shaker for a further 5 min, and then the visual check repeated. Under optimal conditions, only small amounts of tissue remained, and the supernatant was very cloudy. No sample was left in digestion buffer for any longer than 15 min, as this strongly reduced cell viability.

After the 15-min digest period, enzyme action was stopped rapidly by adding 35 ml of ice-cold RPMI 1640 (10% FCS, 100 U/ml Penicillin, 100μg/ml Streptomycin and 2 mM l-glutamine) to each tube, and placing on ice. Each sample (all 50 ml) was then filtered through a 100 μm nylon mesh filter, followed by a 40 μm nylon mesh filter, using a 25 ml stripette and several 50 ml centrifuge tubes. It was important not to crush through any tissue remaining on the top of the filters, as dying connective tissue decreased the viability of isolated cells. The samples were spun at 400 *g* for 10 min at 4 °C to pellet the isolated cells. The supernatants were discarded, and the pellets gently resuspended in 35 ml of ice-cold RPMI 1640 (10% FCS, 100 U/ml Penicillin, 100μg/ml Streptomycin and 2 mM l-glutamine). The spin and resuspension of the pellet was repeated once more, as a final wash, and the resulting cell suspension was counted using trypan blue and kept on ice, ready for further analysis. For maximal cell viability, it should take no longer than 3 to 3.5 h from animal euthanasia to obtaining a single cell suspension.

### 2.3 Key points for successful LP isolations

1. Speed and efficiency, to minimise time from euthanasia to final cell fixation
2. Ensuring effective removal of mucus during processing
3. Being efficient between EDTA washes – do not let tissue dry out at any stage
4. Ensuring all buffers are at correct temperatures
5. Add collagenase VIII to RPMI 1640 immediately prior to use, and dissolve gently
6. Keep samples on ice where possible
7. Do not crush remaining tissue through strainers after digestion
8. Ensure centrifugation steps post-digestion are 10 min long, to maximise cell yield

### 2.4 T cell cytokine stimulation and intracellular staining

Cells were resuspended at 3 × 10^^6^ cells/ml and 1 ml of cell suspension was spun down at 400 *g* for 5 min at 4 °C in FACS tubes. Samples were resuspended in 500 μl of RPMI 1640 supplemented with 10% FCS, 100 U/ml Penicillin, 100μg/ml Streptomycin and 2 mM l-glutamine and 2 μl/ml solution of both stimulation cocktail and protein transport inhibitors (Invitrogen eBioscience™ Cell Stimulation Cocktail plus protein transport inhibitors (500×)). Samples were incubated for 4 h at 37 °C in a 5% CO2 incubator, and vortexed every hour. Samples were then washed twice with cold PBS before staining for flow cytometry. Dead cells were excluded using Fixable Viability Dye eFluor 780 (Ebioscience) and non-specific binding was blocked with Fc block anti-mouse CD16/32 Antibody (Clone 93, BioLegend). Samples were surface stained at 4 °C for 20 min with: APC-conjugated anti-CD4 (RM4–5, BioLegend), FITC-conjugated anti-CD44 (IM7, BioLegend), and PerCpCy5.5-conjugated anti-TCRβ (H57–597, BioLegend). Cells were fixed in 150 μl of BD Cytofix/Cytoperm™ (554714) for 20 min at 4 °C. Samples were then washed using BD Perm/Wash™ Buffer (554714) and 50 μl of intracellular anti-cytokine antibody stain (PE-Cy7-conjugated anti-IL-13 (eBio13A, Invitrogen), PE-conjugated anti-IL-5 (TRFK5, BioLegend), e450-conjugated anti-IFNγ (XMG1.2, Invitrogen)) or appropriate isotype control added to each sample and incubated at room temperature, protected from light, for 1 h. Samples were washed using BD Perm/Wash™ Buffer and acquired immediately on the BD LSRII flow cytometer running FACS-Diva software (BD Biosciences). Analysis was performed using FlowJo (Treestar).

### 2.5 Myeloid cell staining and flow cytometry

For myeloid cell staining, dead cells were excluded using Fixable Viability Dye eFluor 780 (Ebioscience) and non-specific binding was blocked with Fc block anti-mouse CD16/32 Antibody (Clone 93, BioLegend). Samples were then surface stained at 4 °C for 30 min with the following mAbs: BV421-conjugated anti-CD45 (30-F11, BioLegend), AlexaFluor 647-conjugated anti-CD3 (17A2, BioLegend), AlexaFluor 647-conjugated anti-B220 (RA3-6B2, BioLegend), AlexaFluor 700-conjugated anti-MHCII (M5/114.15.2,BioLegend), PerCP-Cy5.5-conjugated anti-CD11c (N418, BioLegend), BV605-conjugated anti-CD11b (M1/70, BioLegend), BV510-conjugated anti-Ly6C (HK1.4, BioLegend), PE-conjugated anti-Ly6G (1A8, BioLegend), and PE-Cy7-conjugated anti-CD64 (X54-5/7.1, BioLegend). Cells were then washed with ice cold PBS (supplemented with 10% FCS and 2 mM EDTA) at 400 g for 10 min at 4 °C and acquired immediately on BD Fortessa flow cytometer running FACS-Diva software (BD Bioscience). Analysis was performed using FlowJo (Treestar). Absolute numbers of cells were calculated by multiplying the total cell count, obtained at the end of section 2.2, with the frequency of the cell population of interest (assessed by flow cytometry).

## 3 Results

**Fig. 1:**
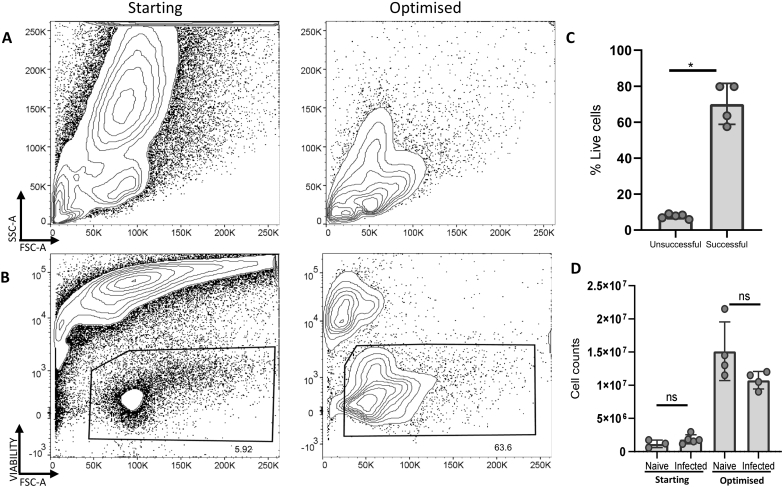
Viability of lamina propria cells isolated using starting and optimised methods. C57BL/6 mice were infected with 200 L3 *H. polygyrus*. 14 days later the small intestine was removed, and LP leukocytes isolated and analysed by flow cytometry. (A) Representative scatter characteristics and (B) representative viability of starting (left) and optimised (right) protocols. (C) % Live cells and (D) Absolute cell numbers of small intestine LP samples from naïve and infected mice, from starting and optimised methods. Graphed data are shown with means ±1 SD and are representative of 3 separate experiments with *n* = 4–5 in each experiment. Statistical significances calculated by Mann Whitney *U* test (Significance *p* < .05, *p < .05).

Several protocols exist for the isolation of LP leukocytes from the small intestine of mice, but the inflamed and mucus-rich conditions of helminth-infected intestines have proven challenging. We set out to optimise isolation conditions for LP leukocytes from the small intestine LP of mice 14 days post-infection with *H. polygyrus*, adapting protocols previously used by the Milling and Mowat lab groups for naïve and bacterially-infected tissues (Chirdo et al., 2005) (Bravo-Blas et al., 2019; Cerovic et al., 2013). Our starting protocols resulted in extensive cell death when processing *H. polygyrus* infected tissues, which we reduced by optimisation of the dissection and digestion workflow and the concentrations of digestion enzymes and EDTA solutions (Fig. 1). We also tried the inclusion of dithiothreitol (DTT) to disrupt mucus, and the use of a density gradient to remove dead cells (Goodyear et al., 2014), but we found that DTT reduced cell viability and the density gradient reduced cell yield. Our optimised protocol removes both steps. When assessing the forward and side scatter of the isolated leukocytes, our optimised protocol showed clear lymphocyte populations that were not present in our previous isolations using our starting protocols (Fig. 1A). Furthermore, when comparing the viability between these experiments, cells isolated using our optimised protocol had much higher viability compared to previous experiments (Fig. 1B and C).

**Fig. 2:**
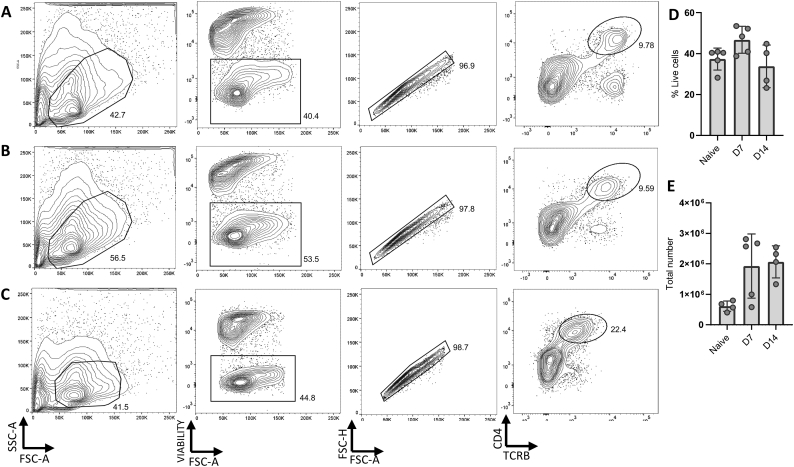
Isolation of CD4^+^ T cells from the small intestine at different stages of *H. polygyrus* infection. C57BL/6 mice were uninfected or infected with 200 L3 *H. polygyrus* larvae. 7 and 14 days later, the small intestine was removed, and LP leukocytes isolated, stimulated with cell stimulation cocktail and protein transport inhibitors, and analysed by flow cytometry. Representative CD4^+^ T cell gating strategy of LP cells from (A) naïve (B) 7 days post-infection and (C) 14 days post-infection. (D) The percentage of live cells in the lymphocyte gate at each timepoint (E) The total number of CD4^+^ T cells at each timepoint. Graphed data are shown with means ±1 SD and are representative of 3 separate experiments with n = 4–5 in each.

We next assessed the effectiveness of our protocol in the isolation of CD4^+^ T cells at different times after *H. polygyrus* infection (Fig. 2). The small intestines of naïve mice or mice 7 and 14 days post-infection with *H. polygyrus* were collected, LP leukocytes were isolated and CD4^+^ T cells subsequently analysed by flow cytometry. Live T cells were recovered at all times after infection (Fig. 2A-C), and the total number of recovered CD4+ T cells showed a trend towards higher yields from infected animals than in naïve, and from day 14 than day 7 (Fig. 2D). The viability of cells remained largely consistent between different timepoints, with a slight decrease at day 14 post-infection (Fig. 2E).

**Fig. 3:**
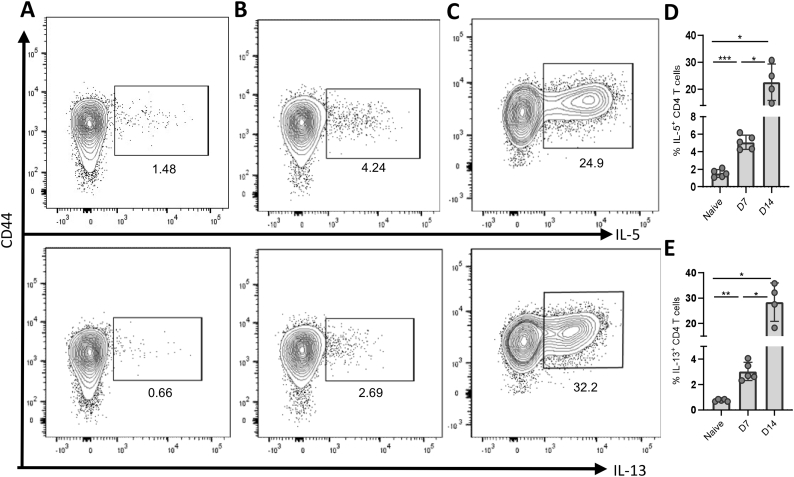
Type 2 cytokine secretion by CD4^+^ T cells during *H. polygyrus* infection. C57BL/6 mice were uninfected or infected with 200 L3 *H. polygyrus* larvae. 7 and 14 days later the small intestine was removed, and LP leukocytes isolated, stimulated with cell stimulation cocktail and protein transport inhibitors, stained for intracellular cytokines and analysed by flow cytometry. Cells shown are gated as CD44^hi^, CD4^+^, TCRβ^+^, single (using FSC-H vs FSC-A), live (fixable viability dye negative) lymphocytes (gated using SSC-A vs FSC-A). Representative CD4^+^ T cytokine secretion of IL-5 (top) and IL-13 (bottom) by LP cells from (A) naïve mice (B) 7 days post-infection and (C) 14 days post-infection. (D) Percentage of IL-5^+^ and (E) IL-13^+^ cells among total CD4^+^ T cells at each timepoint. Graphed data are shown with means ±1 SD and are representative of 3 separate experiments with *n* = 4–5 in each experiment. Statistical significances were calculated by Brown-Forsythe ANOVA test followed by Dunnett's T3 test for multiple comparisons between groups with different standard deviations (**p < .01 *p < .05).

**Fig. 4:**
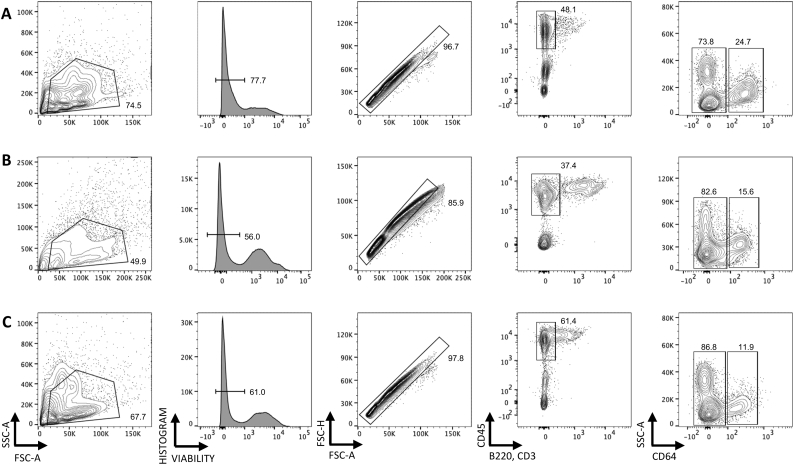
Isolation of myeloid cells from the small intestine of *H. polygyrus* infected mice. C57BL/6 mice were uninfected or infected with 200 L3 *H. polygyrus* larvae and 7 and 14 days later the small intestine was removed and LP leukocytes isolated and analysed by flow cytometry. Representative myeloid cell gating strategy of LP cells from (A) naïve (B) 7 days post-infection and (C) 14 days post-infection. Data representative of 3 separate experiments and n = 4–5 in each experiment.

To assess the function of recovered CD4^+^ T cells, we next examined effector subsets based on cytokine secretion. This technique requires a 4-h stimulation with PMA and ionomycin in the presence of Golgi inhibitors. This is a harsh stimulation and it often decreases cell viability, even in cells from easily accessible tissues. In our previous isolation protocols, cells isolated from the small intestine of *H. polygyrus* infected mice showed almost complete cell death after stimulation. To test the current method, we stimulated bulk cells and stained both for the surface markers CD4 and TGFβ and, intracellularly, for the type 2 cytokines IL-5 and IL-13. Live cells were recovered (Fig. 2D-E, Fig. 3A-E), and cytokines were detected at all time points post-infection (Fig. 3A-C, D, E). As predicted, IL-5 and IL-13 positive cells were more frequent in intestinal samples from infected mice compared to those from naïve controls (Fig. 3A-E). Isolating larger immune cells such as myeloid cells from *H. polygyrus* infected small intestines has also been difficult previously. These cells are typically less robust than CD4^+^ T cells in surviving the isolation process, and the extent of tissue digestion must be finely balanced between sufficient to release adherent cells but gentle enough that cell viability is preserved. We used our optimised protocol to examine the myeloid compartment in the small intestine at different times after *H. polygyrus* infection (Fig. 4).

**Fig. 5:**
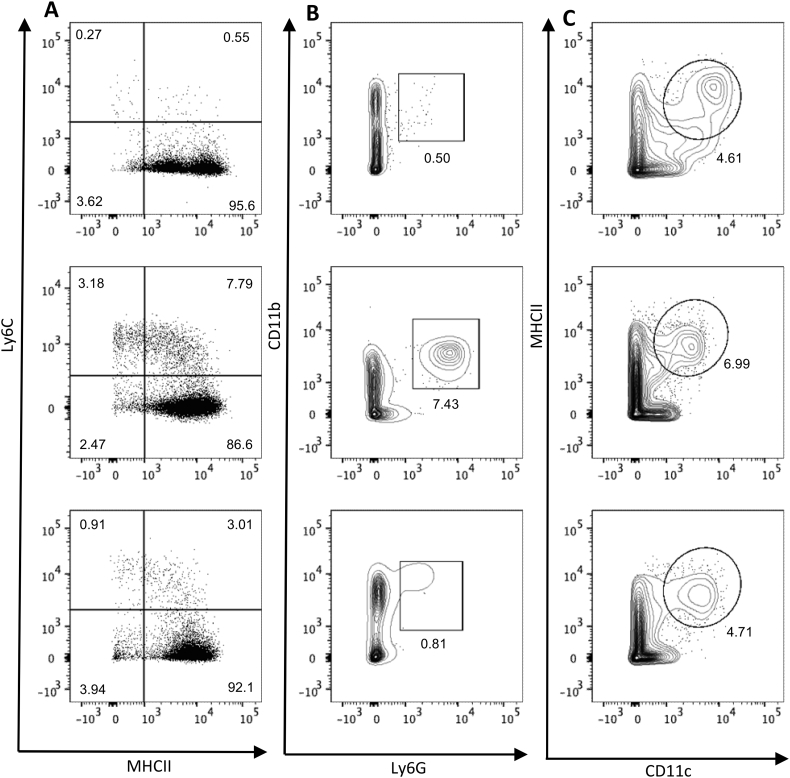
Isolation of monocytes, macrophages, neutrophils and dendritic cells from small intestinal LP at different stages of *H. polygyrus* infection. C57BL/6 mice were uninfected (left) or infected with 200 L3 *H. polygyrus* larvae and 7 (middle) and 14 (left) days later the small intestine was removed and LP leukocytes isolated and analysed by flow cytometry. (A) Monocytes (MHCII^−^, Ly6C^+^), maturing monocytes (MHCII^+^, Ly6C^+^) and macrophages (MHCII^+^, Ly6C^−^) isolated at different time points of infection. Gated on live, single, CD45^+^ B220^−^ CD3^−^ CD64^+^ cells. (B) Neutrophils (Ly6G^+^, CD11b^+^) isolated at different time points of infection. Gated on live, single, CD45^+^, B220^−^ CD3^−^, CD64^−^ cells. (C) Dendritic cells (MHCII^+^, CD11c^+^) isolated at different time points of infection. Gated on live, single, CD45^+^ B220^−^ CD3^−^ CD64^−^ cells. Data are representative of 3 separate experiments with n = 4–5 in each experiment.

**Fig. 6:**
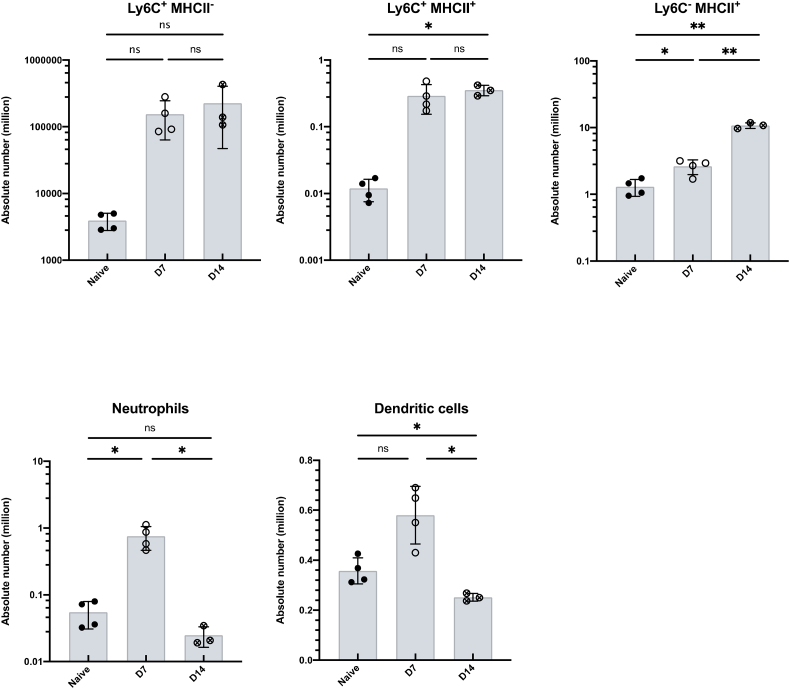
Absolute numbers of monocytes, macrophages, neutrophils and dendritic cells recovered from small intestinal LP at different stages of *H. polygyrus* infection. C57BL/6 mice were uninfected or infected with 200 L3 *H. polygyrus* larvae and 7 and 14 days later the small intestine was removed, and LP leukocytes isolated and analysed by flow cytometry. (A) Absolute numbers of monocytes (MHCII^−^,Ly6C^+^), maturing monocytes (MHCII^+^, Ly6C^+^) and mature macrophages (MHCII^+^, Ly6C^−^) isolated at different time points of infection. Gated on live, single, CD45^+^, B220^−^ CD3^−^, CD64^+^ cells. (B) Absolute number of neutrophils (Ly6G^+^, CD11b^+^) isolated at different time points of infection. Gated on live, single, CD45^+^, B220^−^ CD3^−^, CD64^−^ cells. (C) Absolute number of dendritic cells (MHCII^+^, CD11c^+^) isolated at different time points of infection. Gated on live, single, CD45^+^, B220^−^ CD3^−^, CD64^−^ cells. Data shown are means ±1 SD and are representative of 2 separate experiments *n* = 3–4. Statistical significances were calculated by ANOVA followed by a Tukey's post-test for multiple comparisons between groups with similar standard deviations (**p < .01, ****p < .0001, ns = non-significant).

The isolated LP leukocytes contained a substantial live cell population that is CD45^+^ but negative for T cell and B cell lineage markers. Using CD64 expression as a marker of monocyte/macrophages that distinguishes these cells from dendritic cell populations in the murine intestine (Cerovic et al., 2014), we confirmed that our protocol can isolate live myeloid cell populations at 14 days post *H. polygyrus* infection (Fig. 4). We also recovered a sufficient number of cells from the infected LP to further analyse distinct subsets of the myeloid lineage (Fig. 5). Following the gating illustrated in Fig. 4 and selecting CD64^+^ cells, we identified infiltrating monocytes (MHCII^−^, Ly6C^+^), maturing monocytes (MHCII^+^, Ly6C^+^) and macrophages (MHCII^+^, Ly6C^−^) (Fig. 5A), illustrated in the ‘monocyte waterfall’ (Bain et al., 2013), at both day 7 and day 14 post infection. Similarly, we identified infiltrating neutrophils (live single CD45^+^ B220^−^ CD3^−^ CD64^−^ Ly6G^+^ CD11b^+^ cells) (Fig. 5B). Finally, we are also able to distinguish dendritic cell populations by gating on live single CD45^+^ B220^−^ CD3^−^ CD64^−^ CD11c^+^ MHCII^+^ cells (Fig. 5C). These strategies suggested increases in the absolute numbers of monocytes (MHCII^−^, Ly6C^+^) and maturing monocytes (MHCII^+^, Ly6C^+^) over the course of *H. polygyrus* infection, although these differences did not reach statistical significance (Fig. 6A). The abundance of neutrophils (Fig. 6B) and dendritic cells (Fig. 6C) did increase at day 7 of infection, before decreasing at day 14. Together these results confirm that the method described here yields high quality cell samples from *H. polygyrus* infected intestinal tissue, that can be further analysed for detailed immune cell subsetting and characterisation.

## 4 Discussion

Our data demonstrate that we have optimised a protocol that allows for successful isolation and functional characterisation of LP leukocytes from the small intestine of *H. polygyrus* infected mice at two key timepoints, day 7 and day 14 post-infection. We were able to identify a clear CD4^+^ Th2 cell population whose frequency and number increased in infection, reflecting the predicted immunobiology of the infection and confirming that, even at day 14 when mucus levels are highly elevated, this protocol is able to recover representative cell populations. Furthermore, we were able to assess cytokine secretion by CD4^+^ T cells from the infected tissue, maintaining adequate cell viability. We also demonstrate that we can isolate distinct populations of monocytes, macrophages, neutrophils and dendritic cells from the small intestine LP of helminth-infected animals, which had previously been a particular challenge due to the fragile nature of myeloid cells ex vivo. Together, our data indicate that the isolation protocol that we present here allows the phenotypic and functional characterisation of innate and adaptive immune cells active in the infected tissue site during a Type 2 immune response to helminth infection.

The isolation of viable leukocytes from the intestinal LP of naïve mice or those infected with bacterial, viral and protozoan pathogens has been published widely (Bravo-Blas et al., 2019; Cerovic et al., 2013; Goodyear et al., 2014; Isakov et al., 2011; Perona-Wright et al., 2012). In contrast, helminth-infected tissues have been difficult to use as sources of immune cells. This is largely attributed to the ‘weep and sweep’ effector mechanisms associated with a type 2 immune response, including extensive mucus production, activation of pro-fibrotic macrophages, lymphocytic and granulocytic infiltration, and the release of histamine and cytokines that lead to a mobile and fragile intestinal epithelium (Allen and Maizels, 2011; Allen and Wynn, 2011; Anthony et al., 2007; Cerovic et al., 2013; Webb and Tait Wojno, 2017). Our understanding of type 2 immunity has therefore been biased towards observations made in the early stages of infection or in lymphoid tissues (Hewitson et al., 2015; Perona-Wright et al., 2010b; Rausch et al., 2010; Redpath et al., 2015; Smith et al., 2018; Su et al., 2018; Wojciechowski et al., 2009). Here, we have optimised the timing, the digestion and the processing of helminth-infected intestinal tissue (See Methods 2.3) and we present a technique that allows immune characterisation of the small intestine LP during an acute type 2 immune response. By aiming to remove rather than add steps to the isolation protocol, we chose to prioritise speed over precision and were able to achieve sample viabilities ranging from 35 to 75% of lymphocytes, even after stimulation with PMA and ionomycin (Figs. 2E and 3). A danger of selecting such a light touch approach was that we might lose cells that were more firmly attached in the inflamed tissue. However, our analysis of myeloid populations (Fig. 4, Fig. 5, Fig. 6) indicates that these cells are also isolated and viable following this protocol. We therefore present a new method suitable for the analysis of both myeloid and lymphoid cells in the LP of mice 14 days after *H. polygyrus* infection.

Our method is complemented by a recent publication by Ferrer-Font and colleagues, which also reports a protocol for isolating live cells from the helminth-infected small intestine LP (Ferrer-Font et al., 2019). The two protocols are similar, despite being developed separately and optimised independently, providing strong cross validation of each other. One difference between the two methods is the enzyme used for tissue digestion: Ferrer-Font et al. selected Collagenase A; we compared a similar range of enzymes and achieved the highest yield and viability with Collagenase VIII.^3^ Collagenase VIII is a mixture of enzymes, including additional proteases such as clostripain, potentially improving the efficacy of digestion. Both protocols have optimised the tissue handling and physical mucus removal in addition to the digestion strength; and both protocols have prioritised speed and gentle handling by removing steps involving DTT and density gradients. Together, the two protocols offer a substantial advance in our ability to analyse effector cell populations from the small intestinal LP during helminth infection.

There is growing interest in the immunobiology of infections at the effector site, rather than associated lymphoid tissues. The characterisation of CD4^+^ and CD8^+^ resident memory T cells in the affected tissues has redefined our models of T cell memory (Carbone and Gebhardt, 2019). T cell cytokines are thought to be segregated by tissue, with IL-4 concentrated in the active lymph node and IL-5 and IL-13 dominating in the infected tissue (Liang et al., 2011; Redpath et al., 2015). Myeloid cells in the effector site are key to tissue remodelling, clearing of microorganisms and debris, and the initiation and perpetuation of appropriate T cell responses (Allen and Wynn, 2011; Kim and Kim, 2018; Rolot and Dewals, 2018). Being able to examine cells and their activation at the tissue site will be critical for understanding the dynamics of the *H. polygyrus* infection model as well as those of a prototypical type 2 immune response. We therefore present a protocol to allow for successful analysis of leukocyte populations at the site of *H. polygyrus* infection, the small intestine LP, throughout the course of infection.

## Footnotes

3 In conversation with Ferrer-Font and colleagues, it took a surprising length of time to realise we use different enzymes, as “A” and “VIII” sound very similar when spoken in New Zealand and Scottish accents, respectively.

## References

[bb0005] Allen J.E., Maizels R.M. (2011). Diversity and dialogue in immunity to helminths. Nat. Rev. Immunol..

[bb0010] Allen J.E., Wynn T.A. (2011). Evolution of Th2 immunity: a rapid repair response to tissue destructive pathogens. PLoS Pathog..

[bb0015] Anthony R.M., Rutitzky L.I., Urban J.F., Stadecker M.J., Gause W.C., Urban J.F. (2007). Protective immune mechanisms in helminth infection. Nat. Rev. Immunol..

[bb0020] Bain C.C., Scott C.L., Uronen-Hansson H., Gudjonsson S., Jansson O., Grip O., Guilliams M., Malissen B., Agace W.W., Mowat A.M. (2013). Resident and pro-inflammatory macrophages in the colon represent alternative context-dependent fates of the same Ly6Chi monocyte precursors. Mucosal Immunol..

[bb0025] Barlow J.L., McKenzie A.N. (2011). Nuocytes: expanding the innate cell repertoire in type-2 immunity. J. Leukoc. Biol..

[bb0030] Bravo-Blas A., Utriainen L., Clay S.L., Kästele V., Cerovic V., Cunningham A.F., Henderson I.R., Wall D.M., Milling S.W.F. (2019). Salmonella enterica serovar typhimurium travels to mesenteric lymph nodes both with host cells and autonomously. J. Immunol. (Baltimore Md.: 1950).

[bb0035] Carbone F.R., Gebhardt T. (2019). Should I stay or should I go—reconciling clashing perspectives on CD4+ tissue-resident memory T cells. Sci. Immunol..

[bb0040] Cerovic V., Houston S.A., Scott C.L., Aumeunier A., Yrlid U., Mowat A.M., Milling S.W. (2013). Intestinal CD103(−) dendritic cells migrate in lymph and prime effector T cells. Mucosal Immunol..

[bb0045] Cerovic V., Bain C.C., Mowat A.M., Milling S.W. (2014). Intestinal macrophages and dendritic cells: what’s the difference?. Trends Immunol..

[bb0050] Chirdo F.G., Millington O.R., Beacock-Sharp H., Mowat A.M. (2005). Immunomodulatory dendritic cells in intestinal lamina propria. Eur. J. Immunol..

[bb0055] Davoine F., Lacy P. (2014). Eosinophil cytokines, chemokines, and growth factors: emerging roles in immunity. Front. Immunol..

[bb0060] Ferrer-Font L., Mehta P., Harmos P., Schmidt A., Price K.M., Hermans I.F., Ronchese F., Le Gros G., Mayer J.U. (2019). Single-cell analysis of intestinal immune cells during helminth infection. bioRxiv.

[bb0065] Gause W.C., Wynn T.A., Allen J.E. (2013). Type 2 immunity and wound healing: evolutionary refinement of adaptive immunity by helminths. Nat. Rev. Immunol..

[bb0070] Gessner A., Mohrs K., Mohrs M. (2005). Mast cells, basophils, and Eosinophils acquire constitutive IL-4 and IL-13 transcripts during lineage differentiation that are sufficient for rapid cytokine production. J. Immunol..

[bb0075] Goodyear A.W., Kumar A., Dow S., Ryan E.P. (2014). Optimization of murine small intestine leukocyte isolation for global immune phenotype analysis. J. Immunol. Methods.

[bb0080] Hewitson J.P., Filbey K.J., Grainger J.R., Dowle A.A., Pearson M., Murray J., Harcus Y., Maizels R.M. (2011). <em>Heligmosomoides polygyrus</em> elicits a dominant nonprotective antibody response directed against restricted glycan and peptide epitopes. The J. Immunol..

[bb0085] Hewitson J.P., Filbey K.J., Esser-von Bieren J., Camberis M., Schwartz C., Murray J., Reynolds L.A., Blair N., Robertson E., Harcus Y., Boon L., Huang S.C.-C., Yang L., Tu Y., Miller M.J., Voehringer D., Le Gros G., Harris N., Maizels R.M. (2015). Concerted activity of IgG1 antibodies and IL-4/IL-25-dependent effector cells trap helminth larvae in the tissues following vaccination with defined secreted antigens, providing sterile immunity to challenge infection. PLoS Pathog..

[bb0090] Isakov D., Dzutsev A., Berzofsky J.A., Belyakov I.M. (2011). Lack of IL-7 and IL-15 signaling affects interferon-γ production by, more than survival of, small intestinal intraepithelial memory CD8+ T cells. Eur. J. Immunol..

[bb0095] Kim B., Kim T.H. (2018). Fundamental role of dendritic cells in inducing Th2 responses. The Korean J. Int. Med..

[bb0100] Liang H.-E., Reinhardt R.L., Bando J.K., Sullivan B.M., Ho I.C., Locksley R.M. (2011). Divergent expression patterns of IL-4 and IL-13 define unique functions in allergic immunity. Nat. Immunol..

[bb0105] Monroy F.G., Enriquez F.J. (1992). Heligmosomoides polygyrus: a model for chronic gastrointestinal helminthiasis. Parasitol. Today.

[bb0110] Mosconi I., Dubey L.K., Volpe B., Esser-von Bieren J., Zaiss M.M., Lebon L., Massacand J.C., Harris N.L. (2015). Parasite proximity drives the expansion of regulatory T cells in Peyer’s patches following intestinal Helminth infection. Infect. Immun..

[bb0115] Mukai K., Tsai M., Saito H., Galli S.J. (2018). Mast cells as sources of cytokines, chemokines, and growth factors. Immunol. Rev..

[bb0120] Pelly V.S., Kannan Y., Coomes S.M., Entwistle L.J., Rückerl D., Seddon B., MacDonald A.S., McKenzie A., Wilson M.S. (2016). IL-4-producing ILC2s are required for the differentiation of T(H)2 cells following Heligmosomoides polygyrus infection. Mucosal Immunol..

[bb0125] Perona-Wright G., Mohrs K., Mayer K.D., Mohrs M. (2010). Differential regulation of IL-4Ralpha expression by antigen versus cytokine stimulation characterizes Th2 progression in vivo. J. Immunol. (Baltimore, Md.: 1950).

[bb0130] Perona-Wright G., Mohrs K., Mohrs M. (2010). Sustained signaling by canonical helper T cell cytokines throughout the reactive lymph node. Nat. Immunol..

[bb0135] Perona-Wright G., Lundie R.J., Jenkins S.J., Webb L.M., Grencis R.K., MacDonald A.S. (2012). Concurrent bacterial stimulation alters the function of helminth-activated dendritic cells, resulting in IL-17 induction. J. Immunol. (Baltimore Md.: 1950a).

[bb0140] Rausch S., Held J., Stange J., Lendner M., Hepworth M.R., Klotz C., Lucius R., Pogonka T., Hartmann S. (2010). A matter of timing: early, not chronic phase intestinal nematode infection restrains control of a concurrent enteric protozoan infection. Eur. J. Immunol..

[bb0145] Redpath S.A., Heieis G., Perona-Wright G. (2015). Spatial regulation of IL-4 signalling in vivo. Cytokine.

[bb0150] Reynolds L.A., Filbey K.J., Maizels R.M. (2012). Immunity to the model intestinal helminth parasite Heligmosomoides polygyrus. Semin. Immunopathol..

[bb0155] Rolot M., Dewals B.G. (2018). Macrophage activation and functions during Helminth infection. Rec. Adv. Lab. Mouse.

[bb0160] Smith K.A., Löser S., Varyani F., Harcus Y., McSorley H.J., McKenzie A.N., Maizels R.M. (2018). Concerted IL-25R and IL-4Rα signaling drive innate type 2 effector immunity for optimal helminth expulsion. eLife.

[bb0165] Su C.W., Chen C.-Y., Li Y., Long S.R., Massey W., Kumar D.V., Walker W.A., Shi H.N. (2018). Helminth infection protects against high fat diet-induced obesity via induction of alternatively activated macrophages. Sci. Rep..

[bb0170] Urban J.F., Katona Im Fau - Finkelman F.D., Finkelman F.D. (1991). Heligmosomoides polygyrus: CD4+ but not CD8+ T cells regulate the IgE response and protective immunity in mice. Exp. Parasitol..

[bb0175] Webb L.M., Tait Wojno E.D. (2017). The role of rare innate immune cells in Type 2 immune activation against parasitic helminths. Parasitology.

[bb0180] Wojciechowski W., Harris D.P., Sprague F., Mousseau B., Makris M., Kusser K., Honjo T., Mohrs K., Mohrs M., Randall T., Lund F.E. (2009). Cytokine-producing effector B cells regulate type 2 immunity to H. polygyrus. Immunity.

[bb0185] Yazdanbakhsh M., Kremsner P.G., van Ree R. (2002). Allergy, parasites and the hygiene hypothesis. Science.

